# The Gap Between Actual and Preferred Allocation of Financial Resources Among Korean Middle-Aged Adults

**DOI:** 10.1093/geroni/igaf122.2411

**Published:** 2025-12-31

**Authors:** Kyungun Lee, Haeun Yoo, Youngwon Nam, Kyungmin Kim

**Affiliations:** Seoul National University, Seoul, Republic of Korea; Seoul National University, Seoul, Republic of Korea; Seoul National University, Seoul, Republic of Korea; Seoul National University, Seoul, Republic of Korea

## Abstract

As a period of “life in the middle” between generations and in the life course, middle-aged adults are required to prepare for their own future as well as respond to family needs across generations. Due to these competing demands, midlife adults often allocate financial resources to various domains in ways that do not align with their preferred priorities—which may influence their current and future well-being. Using a sample of 2,465 married middle-aged individuals who have at least one child and one living parent (aged 51–59) from the 2014 Korean Baby Boomer Panel Study, we examined actual and preferred ratios of financial resources allocated to (a) current life, (b) retirement preparation, (c) offspring support, and (d) caring for parents. On average, the actual ratios of resources allocated to current life (48% vs. 42%) and offspring support (24% vs. 20%) were higher than preferred ratios (i.e., over-allocation), whereas the actual ratios allocated to retirement preparation (22% vs. 30%) and caring for parents (6% vs. 8%) were lower than preferred ones (i.e., under-allocation). The gaps between actual and preferred allocation to each domain revealed significant associations with life satisfaction of respondents. Over-allocation to current life and under-allocation to retirement preparation were related to lower life satisfaction. Over- and under-allocation to offspring were both related to lower life satisfaction, whereas under-allocation to parents was related to lower life satisfaction. Our findings suggest the importance of balanced allocation of financial resources across domains in understanding well-being of midlife adults.

